# The mediating role of knowledge transfer in the relationship between transformational leadership and digital healthcare performance in the Eastern Health Cluster, Saudi Arabia

**DOI:** 10.1186/s12913-026-14425-1

**Published:** 2026-04-07

**Authors:** Reem Mohammed Abutaleb, Idris Adewale Ahmed, Dhakir Abbas Ali

**Affiliations:** Faculty of Applied Science, Lincoln University College, Kelana Jaya, Petaling Jaya, Selangor 47301 Malaysia

**Keywords:** Transformational leadership, Knowledge transfer, Digital healthcare performance, PLS-SEM, Eastern Health Cluster, Saudi Arabia

## Abstract

Digital healthcare performance is a central objective of Saudi Arabia’s Vision 2030 reforms, particularly within the Eastern Health Cluster, where substantial investments in electronic health records, telemedicine, and other platforms require evidence of organisational impact. Transformational leadership and knowledge transfer are viewed as key drivers of digital performance, yet their combined effects remain under examined in integrated Gulf health systems. This study investigates whether knowledge transfer mediates the relationship between transformational leadership and digital healthcare performance in the Eastern Health Cluster, while accounting for change management practices. A quantitative cross-sectional survey was administered to employees across clinical, managerial, and digital roles using stratified random sampling (*n* = 402). A 40 item Likert scale instrument captured transformational leadership, change management, knowledge transfer, and digital healthcare performance. Partial least squares structural equation modelling with bootstrapping was used to assess the measurement and structural models and to test mediation. The model explained 37.7% of the variance in knowledge transfer (R² = 0.377) and 62.5% of the variance in digital healthcare performance (R² = 0.625). Transformational leadership and knowledge transfer both had significant positive paths to digital healthcare performance, and knowledge transfer partially mediated the leadership effect (indirect β = 0.181, *p* < 0.001; direct β = 0.321, *p* < 0.001). These findings position knowledge transfer as a critical conduit through which leadership behaviours shape digital outcomes and suggest that leadership development under Saudi digital health reforms should be coupled with deliberate knowledge management strategies in integrated care clusters.

**Supplementary Information:**

The online version contains supplementary material available at 10.1186/s12913-026-14425-1.

## Introduction

### Background and context

Digital transformation is now a fundamental health system approach to greater efficiency and responsiveness to increasing demands of safe and prompt care. Digital health is established as one of the pillars of a more efficient, patient centred system in vision 2030 and the Health Sector Transformation Program with investments in electronic health records, telemedicine, virtual self-care, and integrated digital platforms. Suleiman and Ming, [39] suggest that reforms in the Vision 2030 strive to transform the system to preventive, patient centred, technology enabled care alongside solving long term access, workforce deficit, and chronic disease burden challenges. Nonetheless, scalability of these technologies has also placed more demands on healthcare organisations to show a quantifiable change in digital healthcare performance, rather than adoption of technology.

Digital healthcare performance in this paper is defined as the level at which digital systems and practices enhance patient outcomes, process efficiency, data quality, coordination and integration, and alignment to organisational objectives. This framing is compatible with the studies on digital intensity that point to the fact that performance relies on the degree to which technologies are incorporated into clinical workflows and management routines rather than the mere presence of technologies [8, 24]. Similarly, Saudi academic medical centre evidence indicates that coordination and access are supported with tools like electronic health records and mobile access, although the realised gains are conditional on successful implementation, work practices, and the abilities of staff [2]. For this reason, digital healthcare performance is treated here as a staff perceived performance construct measured reflectively, because the observed indicators are viewed as manifestations of an underlying capability experienced across routine clinical and operational work rather than independent components that simply add up.

The Eastern Health Cluster (EHC) offers a practical empirical backdrop since this is a built-in delivery system established through Health Holding Company and actively developing digital platforms and enhancing accountable care objectives. EHC provides medical care to approximately 1.9 million beneficiaries in 120 primary care centres, a medical city, and 22 general and specialty hospitals with a total capacity of 3,456 beds in the Eastern Province [14]. This organisational diversity and scale make EHC an appropriate environment to study the influence of leadership and knowledge processes on the performance of digital healthcare in practice.

### Problem statement and research gap

Transformational leadership is very well known as a force of organisational change, innovation and embracing technology. Transformational leaders may be more effective in enhancing organisational performance by influencing strategy, enhancing learning cultures, and influencing the sharing of knowledge [37]. Transformational leadership in service organisations assists learning and innovation by enabling knowledge management in terms of acquisition, transfer and application [12]. Transformational leadership has been associated with improved performance by physicians and nurses in Saudi hospitals, especially due to behaviours like idealised influence, intellectual stimulation, and individual consideration (Mughal, [30]). More recent work, including Alwali and Alwali [9] and Kludacz Alessandri et al. [24] also points to the need for leadership behaviours that fit digital contexts if technology investments are to translate into performance gains.

Despite this evidence, two gaps remain that are directly relevant to Saudi Arabia’s digital reform agenda. First, much of the leadership literature focuses on financial, innovation, or general organisational performance, rather than a multidimensional construct of digital healthcare performance that captures patient, process, data, and integration outcomes in real delivery systems. Second, mechanisms linking transformational leadership to digital performance remain under specified in healthcare, particularly mechanisms grounded in knowledge processes. Evidence outside Saudi healthcare shows that knowledge transfer can partially mediate relationships between transformational leadership and innovation outcomes [34, 37], and recent work in other national settings indicates that leadership can strengthen knowledge acquisition and transfer through behavioural and work design pathways [40].

Nevertheless, there is still a paucity of similar models of mediation that place knowledge transfer in the relationship between the transformational leadership and digital healthcare performance in the gulf or Saudi healthcare organisations. In this regard, the gap herein discussed is more empirical and contextual, whereby the research can determine whether the established leadership and knowledge mechanisms work in the same manner in an integrated Saudi health cluster.

This disparity is significant since Vision 2030 changes are aimed at not only digitising services but also developing learning organisations that can maintain the improvement. In the absence of empirical data on the interaction between leadership and knowledge transfer to influence digital healthcare performance in integrated clusters like EHC, managers and policymakers have little data on where to invest in leadership development, knowledge management, and implementation support.

### Research question and objectives

The study addresses the following central question:

**Does knowledge transfer mediate the impact of transformational leadership on digital healthcare performance in the Eastern Health Cluster in the Kingdom of Saudi Arabia?**

To address this question, the study adopts a quantitative cross-sectional design and a PLS-SEM analytical approach, using validated scales for transformational leadership, knowledge transfer, and digital healthcare performance. While the broader doctoral project also considers change management and other determinants, this article concentrates on the transformational leadership pathway and the mediating role of knowledge transfer, consistent with evidence that leadership behaviours strongly shape knowledge processes in digitally transforming organisations [12, 40]. The specific objectives are:

1. To assess the direct effect of transformational leadership on digital healthcare performance in the Eastern Health Cluster.
2. To test the effect of transformational leadership on knowledge transfer among healthcare professionals in the cluster.
3. To estimate the effect of knowledge transfer on digital healthcare performance.
4. To evaluate the mediating role of knowledge transfer in the relationship between transformational leadership and digital healthcare performance.

### Contribution and article structure

This article makes three contributions. First, it empirically supports a transformational leadership to knowledge transfer to digital healthcare performance pathway in Saudi healthcare. Second, it generalizes the transformational leadership research to staff perceived digital healthcare performance as opposed to overall performance outcomes. Third, it incorporates evidence of integrated health clusters that work within the context of Vision 2030 reforms that are still underrepresented in the literature. This model is construed as a partial mediation that is, knowledge transfer is one significant means through which the direct effects of leadership can be enhanced and that the larger change environment context can also be prevailed by it.

The remainder of the article is structured as follows. Chapter 2 reviews relevant literature and develops hypotheses. Chapter 3 presents the methods. Chapter 4 reports the results. Chapter 5 interprets findings in relation to prior research and the Saudi policy context. Chapter 6 outlines implications, limitations, and directions for future research.

## Literature review and hypotheses

### Digital healthcare performance

Digital healthcare performance (DHP) can be defined as the degree to which digital technologies enhance care quality, efficiency, safety, coordination, and patient experience, in an organisation. The literature on digital transformation emphasizes the fact that technologies like electronic health records, telemedicine, and remote monitoring can only bring value into play when they transform clinical and operational routines, but not digitising inherent inefficiencies ([2]; Kludacz Alessandri et al., 2025). This difference is important because in most health systems there are high investment levels in digital tools that are not matched by consistent performance improvement, suggesting that socio technical factors drive performance and not technical ones.

A similar term is the digital intensity which is defined as the scope of organisational areas facilitated by digital solutions such as documentation, scheduling, communication, decision support, and analytics (Kludacz Alessandri et al., 2025). Digital intensity can be used to define coverage, but it does not always mean performance. Organisations are able to digitalise a variety of functions and are yet to grapple with disjointed workflows, poor interoperability, or even low levels of clinical adoption. This implies that there is a conflict between adoption indicators and the ability-based results and it is desirable to research on the concept of DHP as a performance construct as opposed to viewing digitalisation as a measure of success.

In Saudi Arabia, Vision 2030 and the Health Sector Transformation Program position digital health as a lever for improving coordination, access, and value [39]. In integrated clusters like the Eastern Health Cluster, performance is determined by whether the digital platforms provide information flow between hospitals and primary care, and population management of chronic disease. In this research, DHP is considered to be a perceived reflective construct of staff. Its logical reasoning is that the indicators are expressions of a deeper capability that is encountered in everyday activity, including higher coordination, higher data utilization, and greater process reliability, as opposed to a collection of separate parts that need to be bundled together to create performance. The approach is in line with leadership and organisational research that evaluates performance with informed perceptions where objective system level indicators are not systematically available or comparable across units.

### Transformational leadership in healthcare and digital transformation

Transformational leadership (TL) is commonly characterised by idealised influence, inspirational motivation, intellectual stimulation, and individualised consideration [4, 15]. The importance of these behaviours in healthcare is that clinical work is multifaceted and values based, and the digital change may jeopardize professional practice and identity. TL can help to align the staff with a common digital vision, justify experimentation, and alleviate the fear of learning new systems, which facilitates implementation.

Empirical research associates TL with performance and innovation. TL correlates with enhanced physician and nurse performance in Saudi hospitals, where inspirational motivation and individualised consideration are advocated to exert effort in high workload (Mughal, [30]). TL facilitates the innovation of products and processes in service organisations by acquiring, transferring, and using knowledge [12]. In other sectors, TL has a more substantial relationship with performance when there is the existence of knowledge sharing cultures [37]. Digitally, digital transformational leadership predicts more digital intensity both directly and indirectly via organisational agility; TL influences the capacity to reconfigure routines around digital tools and not just adoption choices [24].

However, a significant part of this literature is detecting statistical impacts without explicitly defining how leadership behaviours are converted into sustained digital performance, which is why the current research has been interested in knowledge transfer as one of the potential mechanisms. There is also supplementary healthcare evidence that leadership allied pathways are significant to transforming the investments in capabilities into performance because AI driven HRM and emotional intelligence enhance performance partly due to the impact of leadership effectiveness [9]. Accordingly, TL is expected to positively influence digital healthcare performance:

#### *H1*

*Transformational leadership has a positive and significant effect on digital healthcare performance.*

### Knowledge transfer in healthcare organisations

Knowledge transfer (KT) can be defined as the transfer and use of knowledge between individuals and units to resolve problems, standardise practice and enhance performance. KT encompasses codified knowledge in protocols and digital systems as well as tacit knowledge based on clinical experience in the healthcare field, but limited by professional boundaries, hierarchy, and complexity of clinical decision-making, where it lies at the core of digital transformation.

Knowledge processes are empirically associated with outcomes. KT partially mediates the TL product innovation relationship and completely mediates the TL process innovation relationship in Bahraini service organisations, suggesting that leadership is frequently translated into results with the help of knowledge processes, not direct control alone [12]. Tacit knowledge exchange and innovation are also strengthened by knowledge sharing that mediates TL and task performance, and learning cultures that tolerate error [26, 27, 34]. These demands are aggravated by the digital transformation, as the quality of implementation requires training, peer learning, and continuous adaptation. Digital tools enhance co-ordination and access in Saudi academic medical centres, yet the benefits are reliant on the quality of implementation and the potential development of staff capabilities [2]. Reforms in Vision 2030 also emphasize the disparities in readiness and skills [39]. The evidence provided by recent studies in public hospitals further connects readiness, digital health transformation, innovative work behaviour, and performance, in support of the notion that behavioural and learning pathways are key towards translating digital initiatives into results [8].

### Linking transformational leadership and knowledge transfer

The concept of transformational leadership is also theoretically linked with knowledge transfer since it can influence both psychological and social circumstances under which sharing can be possible. Transformational leaders can make organizations more open, decrease defensiveness, and justify time to learn by making goals clearer, acknowledging contributions and encouraging staff to break routine. Such conditions are indispensable in health care where status disparities and risk issues can become an obstacle to knowledge exchange.

This correlation has empirical support. Transformational leadership has been reported to enhance knowledge sharing by enhancing team goal commitment and identification [1, 28]. It has also been discovered that climates of learning, friendship, and psychological safety reinforce knowledge exchange and innovative behaviour, which means that leadership can modify knowledge transfer via team climate and relational conditions [21, 26, 27]. This is in tandem with the general perception that transformational leadership develop learning-oriented cultures in which tacit knowledge is easily shared.

The leadership-to-knowledge connection in digital transformation programmes should be manifested in the form of practical interventions, which may include sponsoring training, facilitating communities of practice, enabling cross functional project teams, and securing time to reflect on implementation issues. Such mechanisms are particularly appropriate in contexts of digital systems in which clinicians and managers have to collectively optimize workflows and map system capabilities into usable routines of practice [32]. Thus, transformational leadership is supposed to elevate the rate and quality of knowledge transfer:

#### *H2*

*Transformational leadership has a positive and significant effect on knowledge transfer.*

### Knowledge transfer and digital healthcare performance

One of the central mechanisms of digital investment being turned into digital healthcare performance is knowledge transfer. The improvement of clinical and operational processes is based on the knowledge of staff on how to record, retrieve, analyze and take action on digital data, and how to collaborate across professional lines using common platforms. The experience of Saudi academic medical centres also shows that the use of digital tools can facilitate coordination and access but requires a structured implementation and improvement of staff capabilities [2]. Saudi-specific evidence on the use of electronic records points to training and literacy as common obstacles to productivity and quality improvements, suggesting that knowledge transfer on how to use systems is not an option but rather a prerequisite [3, 6].

In addition to the initial learning, transfer of knowledge also facilitates the diffusion of the best practices among early adopters, standardisation across sites, and minimisation of variability in the execution of workflows. The reviews of the digital health adoption in Saudi Arabia also indicate the presence of organisational and human factors that determine whether digital applications have a positive impact on workflow and outcomes, which corroborates the thesis that peer learning and shared sensemaking are performance relevant [7]. Knowledge transfer is even more expected to be performance critical in integrated clusters since interoperability across facilities among others needs consistency in use to maintain continuity of care and population level management [22]. Knowledge transmission also facilitates the refinement of workflows in an iterative manner and safer choices since teams learn by errors and develop processes and minimize redundancy [16]. Accordingly:

#### *H3*

*Knowledge transfer has a positive and significant effect on digital healthcare performance.*

### Knowledge transfer as a mediator between transformational leadership and digital healthcare performance

The preceding sections imply that transformational leadership and knowledge transfer operate jointly rather than as separate levers. Transformational leadership can influence digital performance directly by prioritising digital initiatives, aligning staff around a digital vision, and reducing resistance to workflow change. Yet leadership is also expected to influence digital performance indirectly by enabling knowledge transfer around new tools and practices, allowing staff to translate leadership intentions into routine level behaviours.

Empirical evidence supports mediation logics of this type. In service organisations, knowledge transfer mediates the relationship between transformational leadership and innovation outcomes, demonstrating that leadership is channelled through knowledge processes [12]. Knowledge sharing has also been shown to mediate transformational leadership effects on task performance and innovative behaviour in other organisational contexts [27, 34]. Related health sector evidence reinforces the broader principle that leadership related mechanisms operate as conduits through which capabilities and technologies translate into performance outcomes [9]. In digital transformation settings, the logic is that leaders influence climates and structures that support learning, and those knowledge flows enable staff to embed digital workflows and improve data use, coordination, and service quality.

In this model, change management is treated as a control to account for broader organisational conditions that can influence both knowledge transfer and digital performance. This addresses internal coherence concerns by clarifying that the focal explanatory pathway remains transformational leadership through knowledge transfer, while acknowledging that structured change initiatives shape the broader implementation environment.

#### *H4*

*Knowledge transfer mediates the relationship between transformational leadership and digital healthcare performance*,* after accounting for change management and relevant contextual factors.*

### Conceptual model

The model proposes that transformational leadership influences digital healthcare performance both directly and indirectly through knowledge transfer. Transformational leadership is expected to increase digital healthcare performance by aligning staff around a digital vision and supporting behavioural and workflow change (H1). Transformational leadership is also expected to strengthen knowledge transfer by promoting learning-oriented climates and structures that enable sharing and application (H2). Knowledge transfer is expected to increase digital healthcare performance by enabling consistent and informed use of digital tools and embedding digital workflows in routine practice (H3). Finally, knowledge transfer is proposed to mediate the relationship between transformational leadership and digital healthcare performance (H4). Change management is included as a control predictor of both knowledge transfer and digital healthcare performance to account for the wider change environment during digital transformation.

**Hypotheses**.

- H1: Transformational leadership has a positive and significant effect on digital healthcare performance.
- H2: Transformational leadership has a positive and significant effect on knowledge transfer.
- H3: Knowledge transfer has a positive and significant effect on digital healthcare performance.
- H4: Knowledge transfer mediates the relationship between transformational leadership and digital healthcare performance.

**Fig. 1:**
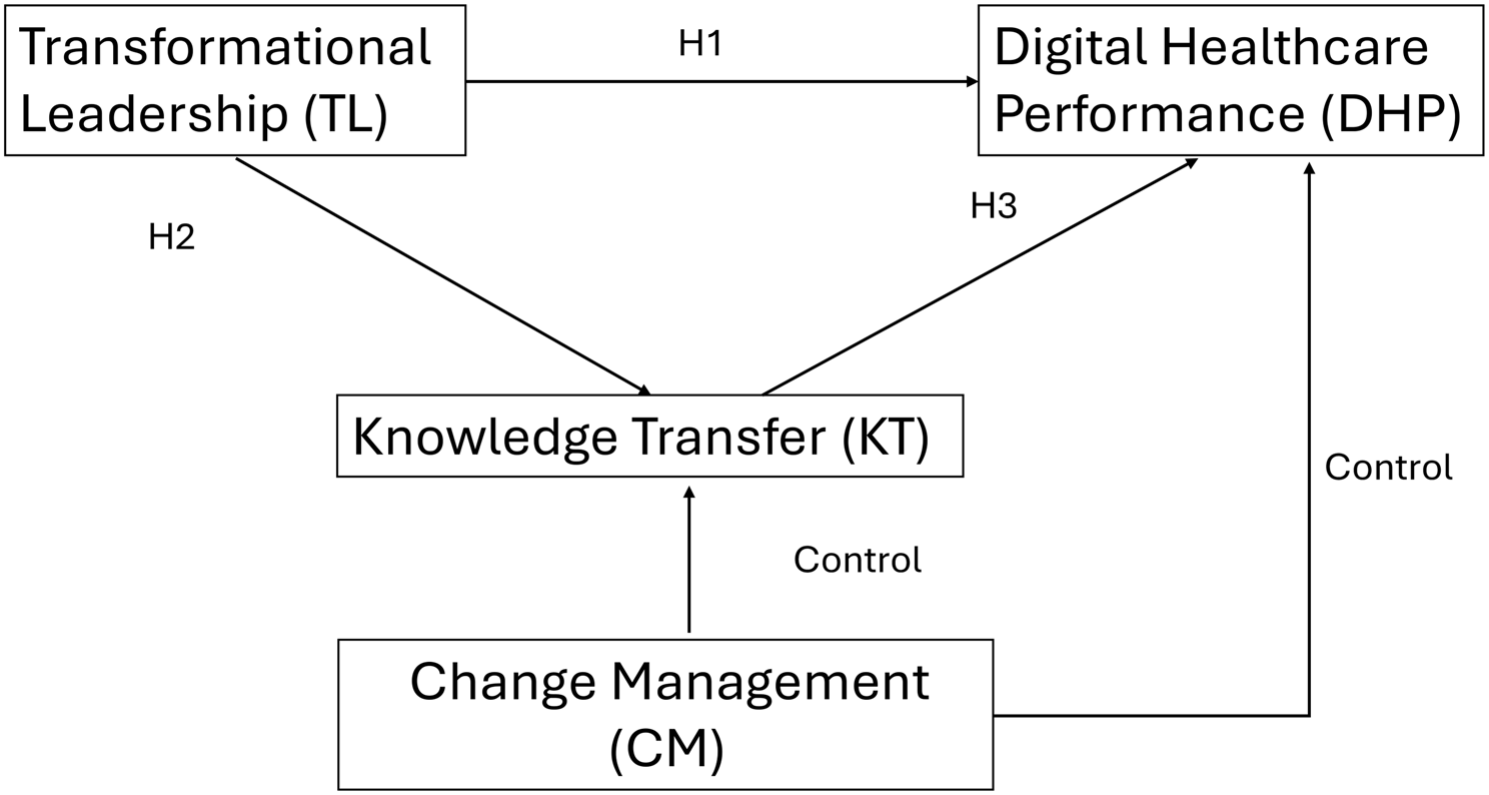
Conceptual model

As shown in Fig. 1, TL is hypothesised to influence DHP directly (H1) and indirectly through KT (H2 and H3), which together constitute the mediation hypothesis (H4). Change management (CM) is included as a control predictor of both KT and DHP.

## Methodology

### Research design

This study employed a quantitative, cross-sectional survey to examine associations among transformational leadership, knowledge transfer, and digital healthcare performance within the Eastern Health Cluster (EHC) in Saudi Arabia. A cross-sectional design was appropriate because the study aimed to estimate relationships among leadership behaviours, knowledge processes, and staff perceived digital performance at a single point during an ongoing transformation stage, rather than to track changes over time. Because the design is cross sectional, it does not support causal inference, and the findings should be interpreted as statistically supported relationships that are consistent with theory but cannot confirm temporal ordering or exclude reverse causality or omitted variables. Given that the conceptual model included multiple latent constructs, a mediating pathway, and change management as a control variable, partial least squares structural equation modelling (PLS SEM) was selected as the primary analytic approach. PLS SEM is well suited to models emphasising prediction and explained variance, particularly where indirect effects are estimated and multivariate normality assumptions may not hold [18, 20]. The use of reflective multi-item scales enabled simultaneous assessment of measurement properties and estimation of structural relationships among constructs within a single integrated model.

### Setting: eastern health cluster and digital health context

The study was conducted in the Eastern Health Cluster (EHC), one of the regional health clusters established under Saudi Arabia’s Vision 2030 health reforms and the Ministry of Health transformation agenda. EHC integrates tertiary and secondary hospitals with primary health care facilities under a shared corporate and clinical governance model intended to strengthen continuity of care, improve resource efficiency, and support population-based service delivery across the Eastern Province. As part of national digitalisation priorities, EHC has implemented major digital health initiatives including electronic health records, telemedicine, mobile health applications, and digital clinical decision support tools, aligning with reforms that emphasise patient centred models of care and data enabled quality and efficiency improvement [5, 38]. This context provides a relevant setting for examining how transformational leadership and knowledge transfer function around digitally supported care and how these organisational dynamics relate to staff perceived digital healthcare performance, with participation spanning clinical staff, digital health specialists, and leadership roles across the cluster.

### Population, sampling, and sample size

The population of interest comprised all employees of the Eastern Health Cluster, estimated at approximately 24,000 staff at the time of data collection. This included clinical staff, digital health professionals, and both clinical and non-clinical leaders. Because the research questions focused on transformational leadership, knowledge transfer, and digital performance, it was important to include respondents who could speak to both frontline experience and leadership and digital roles.

A stratified random sampling strategy was employed. The sampling frame was divided into four strata: clinical staff, health informatics and digital health staff, clinical leaders, and non-clinical leaders. Equal allocation was used at the invitation stage to prevent small strata, particularly digital health staff and leaders, from being under represented in the achieved sample. However, equal allocation produces a disproportionate sample relative to the EHC workforce structure. To restore representativeness at the analysis stage, post stratification weights were applied. Weights were calculated as the ratio of each stratum’s population proportion to its sample proportion, using the population estimates supplied by EHC HR reports at the time of the study. All structural model estimates were therefore computed using weighted data, and unweighted estimates were retained as a robustness check to confirm that the substantive conclusions were not driven by the weighting procedure.

Sample size was determined using two complementary approaches. First, the Krejcie and Morgan approach for finite populations indicated a minimum required sample of approximately 378 for a population of 24,000 at a 95% confidence level and a 5% margin of error. Second, given the use of SEM with mediation and multiple latent variables, a target of 400 completed questionnaires was set to strengthen estimation stability and statistical power, consistent with SEM guidance that samples between 200 and 400 are typically adequate for models of this complexity [19, 23]. Third, to account for non-response observed during piloting, invitations were inflated by 15%, resulting in 460 invitations.

$$\:n=\:\frac{{\chi\:}^{2}Np(1-p)}{{e}^{2}\left(N-1\right)+{\chi\:}^{2}p(1-p)}$$

Where *n* = the estimated sample size, *N* = Population size, *e* = margin of error, which is generally 0.05 or 5%, *p* = proportion of the population, which is normally assumed to be 0.5 as this value provides maximum sample size, and $\:{\chi\:}^{2}$ is the chi square value for the selected confidence level. To account for expected non response and unusable questionnaires, the initial sampling target was inflated to 460 invited participants, producing 115 invitations per stratum. The survey link was sent via official EHC email addresses. A total of 407 questionnaires were returned. Five responses were excluded due to substantial missing data or clear straight line response patterns. The final analytic sample comprised 402 usable questionnaires, representing an effective response rate of 88.47%. Table 1 below summarises the sampling frame, invitations, and usable responses by stratum.

**Table 1:** Structural model and mediation results

Stratum	Population estimate	Invited	Returned	Usable
Clinical staff	14,000	115	100	99
Health informatics / digital health staff	2,000	115	98	95
Clinical leaders	3,000	115	105	103
Non clinical leaders	5,000	115	104	105
**Total**	**24**,**000**	**460**	**407**	**402**

(Population figures are approximate, provided by EHC HR reports at the time of the study

Post stratification weights were computed as $\:{w}_{h}=({N}_{h}/N)/({n}_{h}/n)$, where $\:{N}_{h\:}$is the population size of stratum $\:h$and $\:{n}_{h}\:$is the achieved sample size of stratum $\:h$. Weighted estimates are presented as primary results because they align the sample back to the known workforce composition.

### Measurement instrument

The questionnaire used in this study was adapted from previously published instruments. An English version of the adapted questionnaire has been uploaded as a Supplementary File.

Data were collected using an online self-administered questionnaire. The instrument contained four main multi-item scales measuring transformational leadership, knowledge transfer, digital healthcare performance, and change management, together with a short section on demographic and professional characteristics. All construct items were measured on a five-point Likert scale ranging from 1 (strongly disagree) to 5 (strongly agree). All items were modelled as reflective indicators of their respective latent constructs.

Instrument development and source transparency: All items were either adopted directly or adapted through contextual rewording to fit the digital transformation environment of an integrated health cluster. A mapping table linking each item code to its original source, and indicating whether it was adopted or adapted, is provided as Supplementary Table S1 (Appendix B).

#### Transformational leadership

Transformational leadership was measured using a 10 item scale (TL1-TL10) adapted from established instruments based on the Multifactor Leadership Questionnaire and related work [11]. Items were reworded to reflect leadership behaviours in a digital transformation context. The scale covered the four classical dimensions of transformational leadership: idealised influence, inspirational motivation, intellectual stimulation, and individualised consideration. Example items included “My direct leader communicates a clear vision for digital health in our organisation” and “My leader encourages us to think about how digital tools can improve patient care.”

#### Knowledge transfer (KT)

KT was assessed with 10 items (KT1-KT10) capturing both formal and informal mechanisms for sharing, disseminating, and applying knowledge about digital healthcare. Items were adapted from validated scales on knowledge sharing and knowledge management processes [12, 28, 40]. The items assessed behaviours such as discussing experiences with digital tools, participating in training, using guidelines and online resources, and helping colleagues solve technology related problems. An example item was “In my unit, employees often exchange tips and experience on how to use digital systems.”

#### Digital healthcare performance (DHP)

DHP was measured using a 10-item scale (DHP1-DHP10) derived from instruments on ICT enabled performance in healthcare and digital intensity in service organisations [2, 24]. The scale captured staff perceptions of the impact of digital tools on clinical quality, process efficiency, data availability, coordination of care, and patient experience. Example items included “Digital systems have improved the timeliness of clinical information at the point of care” and “Digital tools help us provide more coordinated and patient centred services.”

#### Change management (CM)

CM practices were assessed with 10 items (CM1-CM10) adapted from existing scales on organisational change management and digital implementation [13, 36]. Items covered communication of change rationales, involvement of stakeholders, availability of training and support, and follow through on change commitments in relation to digital projects. An illustrative item was “Management provides clear information about why digital changes are being introduced.” Change management was specified as a control variable and secondary predictor of knowledge transfer and digital healthcare performance in the structural model.

Because the study was conducted in an Arabic speaking context, the original English items were translated into Arabic using a forward translation and back translation procedure. A bilingual health services researcher translated the items into Arabic, and an independent translator back translated them into English. Discrepancies were discussed and resolved to ensure semantic equivalence.

Content validity was assessed by asking 10 representatives of the target population, drawn from different EHC professional groups, to review the questionnaire for clarity, relevance, and completeness. Their suggestions led to minor wording adjustments and reordering of some items to improve flow. Two survey methodology experts also reviewed the instrument for structure and readability. A pilot test with a small sample of EHC staff was then conducted to check completion time and initial reliability. All scales showed acceptable internal consistency in the pilot, with Cronbach’s alpha coefficients above 0.70, and no items were removed at this stage.

### Data collection procedures and ethical approval

Data were collected using an online self-administered questionnaire. Selected staff received an email invitation through their official Eastern Health Cluster accounts containing a participant information sheet and a unique survey link. The information sheet explained the study purpose, voluntary participation, estimated completion time (approximately 15 min), and assurances of anonymity and confidentiality. No incentives were provided. Data collection remained open for two weeks, with one reminder email issued after seven days. The survey settings permitted one submission per invitee.

Ethical approval was granted by the Institutional Review Board at the Research Center, King Khalid Medical City (RC KKMC), King Fahad Specialist Hospital, Dammam (reference CLU0030; approval period 09/03/2025 to 08/03/2026). The IRB approved a waiver of signed written consent on the basis that an approved information sheet would be provided and that questionnaire completion would constitute implied consent. Participants were informed that they could discontinue at any point before submitting their responses and that no personally identifying information would be collected. Data were stored on password protected servers accessible only to the research team and findings were reported in aggregate form only. All procedures followed Saudi National Committee of Bioethics regulations and were consistent with the Declaration of Helsinki and good clinical practice principles.

### Data preparation and screening

After survey closure, data were exported to IBM SPSS Statistics (version 23) for cleaning and preliminary screening. Each record was assigned a numeric identifier. Five responses were removed due to substantial missing data or clear straight line response patterns. The final analytic dataset included 402 usable cases. Because item responses used a five-point Likert scale, extreme univariate outliers were unlikely; nonetheless, frequency tables and boxplots were inspected and no additional cases were removed. Descriptive statistics (means, standard deviations, skewness, and kurtosis) were computed for all items.

Multivariate normality was assessed using Mardia’s coefficients. The multivariate skewness was 98.6 and kurtosis was 237.6, with p values below 0.05, indicating non normality. This supported the use of PLS SEM with bootstrapping rather than covariance-based SEM approaches that rely more strongly on distributional assumptions [23, 29].

### Statistical analysis strategy

Analysis proceeded in four stages. First, descriptive statistics were generated in SPSS for demographic variables and construct indicators, and Pearson correlations were computed to examine bivariate associations among transformational leadership, knowledge transfer, digital healthcare performance, and change management.

Second, exploratory factor analysis was conducted in SPSS to provide initial evidence that the measurement items clustered into the four theorised constructs. Sampling adequacy and factorability were examined using the Kaiser Meyer Olkin statistic and Bartlett’s test of sphericity. Principal components extraction with Varimax rotation was applied, with loadings below 0.40 suppressed. The rotated factor loadings are reported in Table 2, and the eigenvalues, percentage variance explained, and cumulative variance are reported in Table 3 in Chap. 4.

Third, the measurement and structural models were estimated using SmartPLS 4.0 (SmartPLS GmbH, Boenningstedt, Germany). PLS SEM was selected because the model includes mediation and multiple endogenous constructs and because the data violated multivariate normality, with estimation focused on prediction and explained variance [19, 35]. Measurement model quality was assessed using indicator loadings, internal consistency reliability (Cronbach’s alpha, composite reliability, and rho A), and convergent validity (average variance extracted). Discriminant validity was evaluated using the Fornell Larcker criterion [17], HTMT ratios, and cross loadings.

Fourth, the structural model was assessed using bootstrapping with 5,000 resamples to test the significance of path coefficients and indirect effects. Explanatory power was evaluated using R² for knowledge transfer and digital healthcare performance, with effect sizes (f²) used to gauge predictor contributions and Q² statistics used to assess predictive relevance. Mediation was tested by estimating the indirect effect of transformational leadership on digital healthcare performance through knowledge transfer and evaluating bootstrapped confidence intervals [31]. Change management was included as a control predictor of both knowledge transfer and digital healthcare performance.

**Fig. 2:**
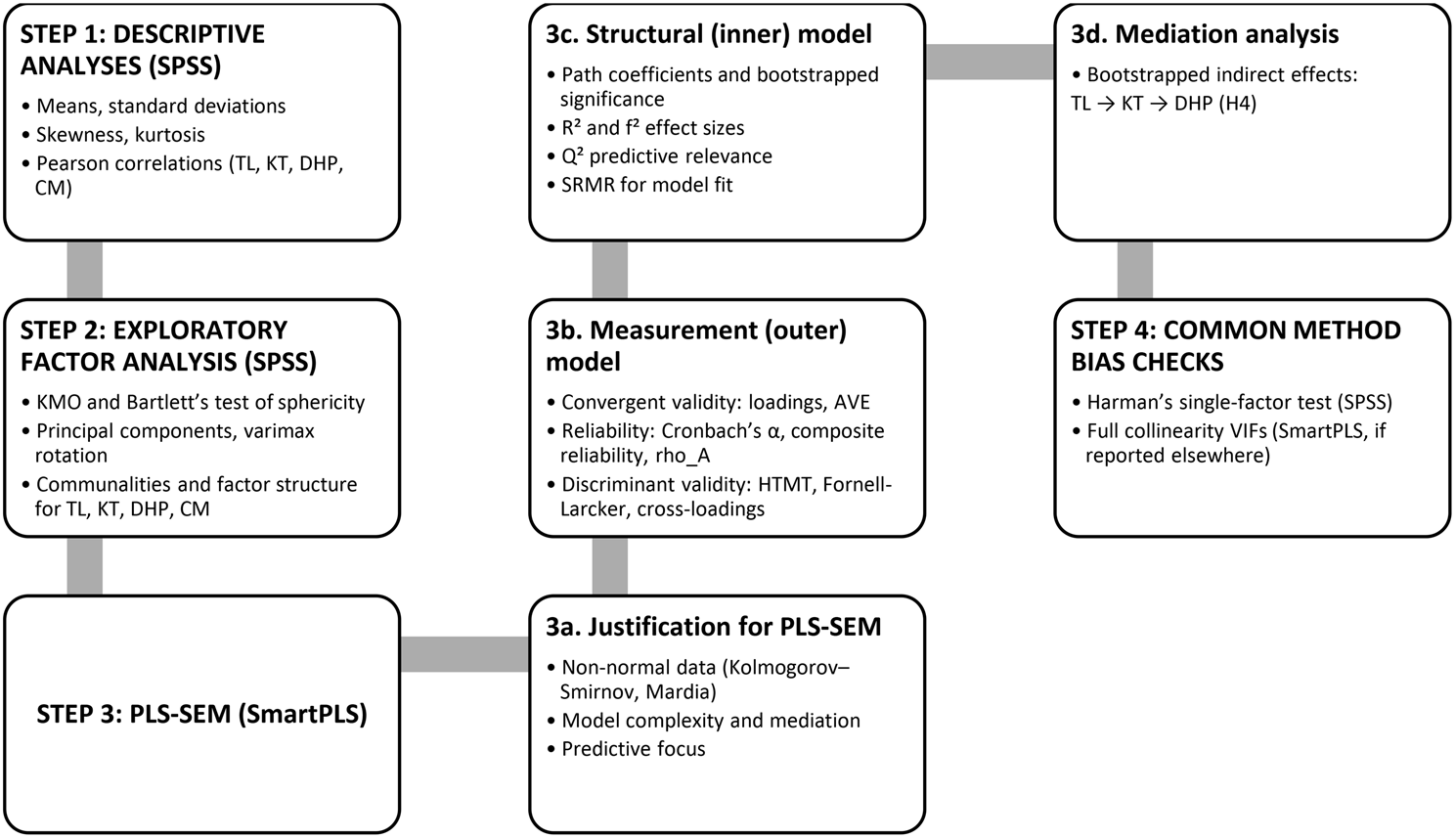
Sequential statistical analysis procedures for testing the conceptual model (SPSS and SmartPLS)

Finally, common method bias was assessed using Harman’s single factor test in SPSS and a full collinearity approach in SmartPLS using variance inflation factors [25]. VIF values were below 5 (CM: 2.63; KT: 1.34; DHP: 1.26), suggesting that common method bias and multicollinearity were unlikely to materially distort the findings [33]. The sequence of analytical procedures, from descriptive statistics and exploratory factor analysis through to PLS-SEM estimation, mediation testing, and common method bias checks, is summarised in Fig. 2 below.

## Results

### Introduction

This chapter presents the empirical results for the study that examined whether KTf mediates the relationship between TL and DHP within the EHC in KSA, while controlling for CM. The analysis follows the sequence set out in Chap. 3. It begins with descriptive results for the sample and key constructs, then reports the measurement model assessment, followed by the structural model, hypothesis testing, and mediation analysis. Results are reported for the study sample (*N* = 402); because the stratified design used equal allocation across strata and no post stratification weights were applied, the estimates should be interpreted as sample based rather than population representative.

### Descriptive results

#### Demographic characteristics of respondents

**Fig. 3:**
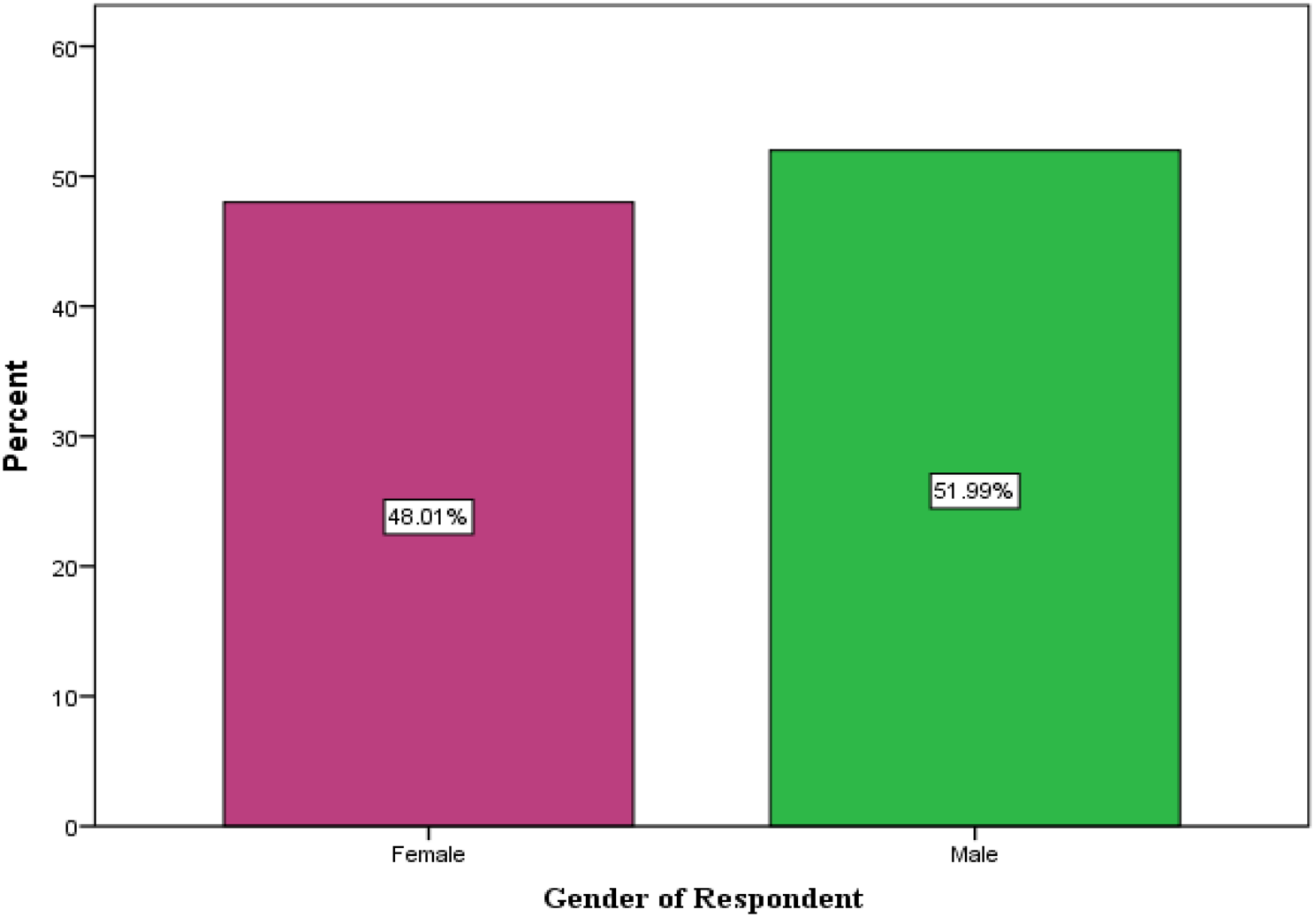
Distribution of respondents by gender

Of the 460 questionnaires emailed to eligible EHC employees, 407 were returned and 5 were removed because of incomplete or straight-line responses, giving a final usable sample of 402 and a response rate of 88.47%. As shown in Fig. 3 below, the demographic profile showed a modest female majority (52%). Age was clustered in the mid-career bands: almost half of respondents (48%) were aged 35–44 years, 21% were 25–34 years, and 25% were 45–54 years, while only 1% fell in the 18–24 group and 4% in the 55–64 group. Educational attainment was high, with 48% holding a bachelor’s degree and 31% a master’s degree. The demographic characteristics of respondents are summarised in the Appendix C.

Job position and experience distributions highlighted the organisational breadth of the sample. Managers (including EHC team leads and hospital administrators) formed the largest single category (38%), with additional representation from clinicians and health informatics professionals (15%), support staff such as technicians (13%), and a range of leadership roles, including department heads, EHC leaders, medical and nursing directors, and quality improvement leaders. Most respondents had substantial professional experience, with 30% reporting 16–20 years in practice and a further 18% more than 20 years.

#### Descriptive statistics for key constructs

Latent construct scores were created by averaging the relevant items for each construct. Descriptive statistics, reliability indices, and inter-construct correlations are presented in Table 2. Additional descriptive statistics for the study constructs are reported in Appendix D. Mean values for TL (M = 3.71, SD = 0.87), CM (M = 3.38, SD = 0.80), KT (M = 3.65, SD = 0.97), and DHP (M = 3.67, SD = 0.80) were all above the neutral point of 3.0 on the five-point scale. Standard deviations were all below 1.0.

**Table 2:** Structural model and mediation results

Construct	Mean	SD	Min	Max	α	CR	AVE	TL	CM	KT	DHP
TL	3.71	0.87	1.40	4.70	0.941	0.950	0.655	1.00	0.63	0.57	0.67
CM	3.38	0.80	1.20	5.00	0.960	0.967	0.745	0.63	1.00	0.52	0.59
KT	3.65	0.97	1.10	5.00	0.950	0.957	0.692	0.57	0.52	1.00	0.71
DHP	3.67	0.80	1.60	4.70	0.920	0.933	0.583	0.67	0.59	0.71	1.00

Note. TL = Transformational leadership; CM = Change management; KT = Knowledge transfer; DHP = Digital healthcare performance. Correlations are Pearson coefficients; all are significant at *p* < 0.001

Descriptive statistics, reliability, and correlations for study constructs (*N* = 402)

### Measurement model

#### Exploratory factor analysis results

An exploratory factor analysis (EFA) was conducted to confirm that the 40 measurement items loaded on the expected four factors (TL, KT, DHP, CM). Kaiser Meyer Olkin sampling adequacy was 0.959 and Bartlett’s test of sphericity was significant (χ² = 14597.07, *p* < 0.001). A four-factor solution accounted for 67.10% of the total variance. The rotated loading matrix is presented in Table 3 and the eigenvalues and explained variance are reported in Table 4. All primary loadings exceeded 0.71 and each item loaded on its intended factor, supporting the expected four construct structure. Cronbach’s alpha values for all constructs are reported in the Appendix E.

**Table 3:** Exploratory factor analysis rotated loadings (Varimax)

Item	Transformational leadership	Knowledge transfer	Digital healthcare performance	Change management
TL1	0.82			
TL2	0.79			
TL3	0.76f			
TL4	0.74			
TL5	0.81			
TL6	0.78			
TL7	0.75			
TL8	0.80			
TL9	0.77			
TL10	0.73			
KT1		0.81		
KT2		0.79		
KT3		0.77		
KT4		0.75		
KT5		0.82		
KT6		0.78		
KT7		0.74		
KT8		0.80		
KT9		0.76		
KT10		0.73		
DHP1			0.79	
DHP2			0.81	
DHP3			0.77	
DHP4			0.75	
DHP5			0.82	
DHP6			0.78	
DHP7			0.74	
DHP8			0.80	
DHP9			0.76	
DHP10			0.73	
CM1				0.80
CM2				0.78
CM3				0.75
CM4				0.82
CM5				0.77
CM6				0.74
CM7				0.79
CM8				0.76
CM9				0.73
CM10				0.71

Note. Extraction: principal components. Rotation: Varimax. Loadings below 0.40 suppressed

**Table 4:** Eigenvalues and explained variance

Factor	Eigenvalue	Percentage of variance	Cumulative percentage
Transformational leadership	9.84	24.6	24.6
Knowledge transfer	6.92	17.3	41.9
Digital healthcare performance	5.71	14.3	56.2
Change management	4.38	10.9	67.1

#### Reliability and convergent validity

Internal consistency reliability was assessed using Cronbach’s alpha and composite reliability (CR). Cronbach’s alpha ranged from 0.920 (DHP) to 0.960 (CM), and CR exceeded 0.93 for all constructs (Table 4.1). Convergent validity was assessed using average variance extracted (AVE). AVE values were 0.745 for CM, 0.583 for DHP, 0.692 for KT, and 0.655 for TL, each exceeding the 0.50 criterion.

#### Discriminant Validity

Discriminant validity was evaluated using heterotrait-monotrait ratio of correlations (HTMT), the Fornell-Larcker criterion. The full Fornell Larcker matrix is reported in Table 5. For each construct, the square root of AVE exceeded its correlations with other constructs. HTMT values ranged from 0.541 to 0.753, all below the conservative 0.85 benchmark, indicating adequate discriminant validity.

**Table 5:** Fornell Larcker criterion (square root of AVE on diagonal)

	TL	CM	KT	DHP
TL	0.809	0.630	0.570	0.670
CM	0.630	0.863	0.520	0.590
KT	0.570	0.520	0.832	0.710
DHP	0.670	0.590	0.710	0.764

### Structural model

#### Direct effects, explained variance, and predictive power

Following confirmation of the measurement model, the structural model was estimated in SmartPLS 4.0 using the PLS algorithm and bootstrapping with 5,000 resamples. TL was specified as the primary predictor, KT as the mediator, and DHP as the focal outcome, with CM included as a covariate predicting both KT and DHP. The coefficient of determination for KT was R² = 0.377, indicating that TL and CM jointly accounted for 37.7% of the variance in knowledge transfer. For DHP, TL, KT and CM together explained 62.5% of the variance (R² = 0.625). Predictive relevance assessed via Q² was 0.366 for KT and 0.497 for DHP. Model fit assessed via SRMR was 0.055.

Structural path estimates and effect sizes are reported in Table 6. All hypothesised direct paths were positive and statistically significant. TL had a significant positive effect on DHP (β = 0.321, t = 4.345, *p* < 0.001) and on KT (β = 0.414, t = 6.199, *p* < 0.001). KT had the strongest direct effect on DHP (β = 0.437, t = 6.507, *p* < 0.001). CM exerted smaller but statistically significant effects on KT (β = 0.262, t = 4.125, *p* < 0.001) and DHP (β = 0.165, t = 2.516, *p* = 0.012). Effect sizes indicated a moderate effect of TL on KT (f² = 0.166), a small to moderate effect of TL on DHP (f² = 0.143), a large effect of KT on DHP (f² = 0.318), and smaller but non trivial effects of CM.

**Table 6:** Structural model and mediation results (Structural model and mediation results (PLS-SEM, *N* = 402)

Path	Type	β	t-value	*p*-value	f²
TL → DHP	Direct	0.321	4.345	< 0.001	0.143
TL → KT	Direct	0.414	6.199	< 0.001	0.166
KT → DHP	Direct	0.437	6.507	< 0.001	0.318
CM → KT	Direct	0.262	4.125	< 0.001	0.067
CM → DHP	Direct	0.165	2.516	0.012	0.041
TL → KT → DHP	Indirect	0.181	4.644	< 0.001	-
CM → KT → DHP	Indirect	0.115	3.361	0.001	-

Note. TL = Transformational leadership; CM = Change management; KT = Knowledge transfer; DHP = Digital healthcare performance. R²: KT = 0.377; DHP = 0.625. Q²: KT = 0.366; DHP = 0.497. SRMR (saturated and estimated models) = 0.055

#### Mediation analysis

The central research question examined whether KT mediates the relationship between TL and DHP. Bootstrapped indirect effects showed that the indirect path TL → KT → DHP was significant (β = 0.181, t = 4.644, *p* < 0.001). The direct effect of TL on DHP remained statistically significant when KT was included (β = 0.321, t = 4.345, *p* < 0.001). The total effect of TL on DHP was 0.502, indicating partial mediation whereby transformational leadership influences DHP both directly and indirectly through knowledge transfer. CM also exhibited a significant mediated pathway through KT. The indirect effect CM → KT → DHP was significant (β = 0.115, t = 3.361, *p* = 0.001), while the direct effect CM → DHP remained significant (β = 0.165, *p* = 0.012), indicating partial mediation for CM as well.

### Additional diagnostics

Prior to estimating the structural model, assumptions about normality and common method bias were checked. Kolmogorov-Smirnov tests showed that construct scores deviated from univariate normality (all *p* < 0.001). Mardia’s coefficients for multivariate skewness and kurtosis (10.703 and 46.77, respectively) were also significant. Common method bias was assessed using Harman’s single-factor test and full collinearity variance inflation factors. The single-factor solution showed that the first factor accounted for only about 18% of the variance, far below the 50% threshold often taken as indicative of serious common method bias. In SmartPLS, full collinearity VIFs for the constructs were all below 5.

### Hypothesis testing summary

Four hypotheses were tested:

- H1: Transformational leadership has a positive and significant effect on digital healthcare performance.
- H2: Transformational leadership has a positive and significant effect on knowledge transfer.
- H3: Knowledge transfer has a positive and significant effect on digital healthcare performance.
- H4: Knowledge transfer mediates the relationship between transformational leadership and digital healthcare performance.

All hypotheses were supported. TL significantly predicted KT and DHP, KT significantly predicted DHP, and the indirect TL → KT → DHP effect was significant while the direct TL → DHP effect remained significant, indicating partial mediation.

## Discussion

This study examined whether knowledge transfer mediates the relationship between transformational leadership and digital healthcare performance in the Eastern Health Cluster in Saudi Arabia, after controlling for change management. Using PLS-SEM with survey data from 402 employees across clinical, digital and leadership roles, all four hypotheses were supported. Transformational leadership had significant positive effects on both digital healthcare performance and knowledge transfer, knowledge transfer significantly predicted digital performance, and the indirect effect of transformational leadership on digital performance through knowledge transfer was significant while the direct effect remained. Taken together, these findings are broadly in line with transformational leadership theory and knowledge-based views of organisational performance [12, 37]. More importantly, they clarify how leadership behaviours and day to day knowledge processes interact within a Saudi cluster that is under pressure to deliver measurable gains from Vision 2030 digital investments [39].

### Transformational leadership and digital healthcare performance

The positive association between transformational leadership and digital healthcare performance (H1) corresponds with prior evidence that transformational behaviours enhance performance and innovation in healthcare and service organisations ([12]; Mughal, [30]). In this study, the path from transformational leadership to digital healthcare performance was significant with a small to moderate effect size. A plausible explanation is that digital performance in a cluster setting depends on coordinated behavioural change, not only on system availability. Leaders who communicate a credible digital vision and link it to clinical values can reduce professional scepticism about whether digitalisation serves patient care or primarily serves reporting and control. In EHC, where digital tools can reallocate tasks, increase documentation burden, and introduce new forms of surveillance, staff are likely to appraise digital reforms through the lens of workload, autonomy, and clinical responsibility. Transformational leaders can buffer these risks by framing change as a shared redesign effort, clarifying what will be simplified, and signalling support when early implementation disrupts routines.

Compared with transactional models that emphasise monitoring and contingent rewards, transformational leadership appears better suited to complex and change intensive contexts. This interpretation is supported by work on digital transformational leadership, which shows that leaders who adapt their behaviours to digital contexts are more successful in mobilising staff and redesigning processes [24]. In the Vision 2030 agenda, where digital health is framed as a lever for access and value, the present results indicate that transformational leadership is a measurable organisational resource that shapes perceived digital performance [39]. However, the modest effect size also suggests leadership alone cannot overcome structural constraints such as system fragmentation, uneven interoperability across facilities, or staffing shortages that intensify cognitive load during digital change.

### Transformational leadership and knowledge transfer

The significant positive relationship between transformational leadership and knowledge transfer (H2) reinforces the idea that leadership shapes the social and psychological conditions under which knowledge is exchanged and used. The path coefficient from transformational leadership to knowledge transfer, with a moderate effect size, suggests that leadership behaviours are a substantive driver of knowledge processes in the Eastern Health Cluster.

As compared with studies in service and knowledge-intensive sectors, where transformational leadership improves knowledge acquisition and transfer through engagement, autonomy and learning climates [12, 32, 34, 40], the current findings suggest that similar dynamics are at work in Saudi healthcare. Affective and motivational perspectives show that transformational leaders raise psychological safety and team identification, making staff more willing to admit knowledge gaps, discuss mistakes and share expertise [10, 28]. Within EHC, leaders who champion digital initiatives, legitimise time for training and peer learning, and encourage cross-functional collaboration appear to foster precisely the conditions in which staff can exchange experiences about digital tools and integrate new knowledge into workflows, thus advancing Vision 2030 ambitions for learning organisations rather than simple digitisation [39].

### Knowledge transfer and digital healthcare performance

Knowledge transfer emerged as the strongest direct predictor of digital healthcare performance (H3), with the largest effect size among the predictors. This finding elaborates the central claim of digital health and knowledge management research that technology investments only yield performance gains when staff are able to interpret digital outputs, adapt routines and coordinate across professional and organisational boundaries [2, 7, 24].

Evidence from Saudi hospitals has repeatedly shown that limited training and inadequate computer literacy undermine the benefits of electronic health records and related applications [3, 6]. The present analysis complements this work by demonstrating that where staff perceive stronger knowledge transfer around digital tools, they also report higher digital healthcare performance. In other words, digital performance seems to depend less on the mere presence of systems and more on how far knowledge about those systems is shared, diffused and embedded in daily practice.

In the EHC context, this matters because digital change often produces uncertainty about correct documentation, new decision support prompts, and revised escalation pathways. When leaders respond to early errors as learning opportunities rather than as blame events, staff are more likely to share practical tips and prevent error repetition.

### Knowledge transfer as a mediator

The mediation analysis showed that the relationship between transformational leadership and digital healthcare performance is partially mediated by knowledge transfer (H4). The indirect relationship between transformational leadership and digital performance through knowledge transfer was substantial, and the direct relationship was still present and gave a large overall effect. Such tendency is quite similar to the findings in non-healthcare organizations where knowledge transfer and application mediate the impacts of transformational leadership on innovation and performance [12, 34, 37].

The partial mediation pattern is theoretically significant as it implies two parallel paths. The direction of one of them is an indication of direct leadership impact via prioritisation, mobilisation of resources, and elimination of barriers to implementation. The second path indicates the indirect impact by channeling knowledge routines that transform leadership intent into routine digital tools use. The findings do not suggest that knowledge transfer represents the leading channel, it merely suggests that knowledge transfer is one of the significant channels with respect to direct leadership effects and the overall change context.

The additional mediation linkage between change management and digital performance through knowledge transfer imply that formal practices including communication, involvement, and training partially work by facilitating knowledge sharing [13, 36]. This favors a socio technical reading whereby, formal change structures establish the environment within which the learning takes place, with leadership defining the quality and credibility of the learning processes.

### Implications for theory

The research has three theoretical contributions. First, it broadens the study of transformational leadership by connecting the concept of leadership to a multidimensional metric of digital healthcare performance in which clinical, process, data, and integration outcomes are aggregated [2, 24]. Second, it supports knowledge based perspectives, indicating that the knowledge transfer is a believable channel through which leadership is linked to the performance outcomes, instead of presuming that the leadership effects act solely via direct oversight or motivation [12, 34, 40]. Third, it validates the external validity of leadership and knowledge mechanisms in Gulf health systems, where digital transformation remains policy driven and institutionally complex under Vision 2030 by testing these relationships in an integrated Saudi cluster [39]. The socio technical context also suggests that leadership and knowledge processes can be particularly salient in the context of digitisation interacting with the demands of rapid service redesign, workforce localisation, and social responsibility.

### Implications for practice and policy

Practically and policy-wise, there are three priorities based on the results. First, the digital and knowledge-oriented competencies should be explicitly included in leadership development. One tangible example of an intervention related to the cluster level is training leaders to facilitate structured digital huddles and debriefs to review documentation bottlenecks, digital incidences, and alert fatigue, and then apply the results of these discussions to agreed workflow changes. Leader coaching must further focus on conveying the message of digital priorities in clinical quality and safety language, rather than efficiency language, because clinicians may be resistant to digitisation when presented as an administrative growth.

Second, digital strategies must invest in non-procurement knowledge transfer infrastructure. It may involve role-based training, communities of practice, early adopters mentorship, and curated repositories, which document local adaptations [2, 7]. These mechanisms can be institutionalized in governance routines, including monthly cross site workflow councils that compare digital metrics and the exchange of implementation fixes, and micro learning modules that alleviate cognitive overload by concentrating on the biggest risks, including medication reconciliation and referral routing.

Third, the implications of change management imply that formal implementation practices support leadership and contribute to learning sustainability. This can be achieved by taking specific actionable steps such as the standardisation of communication packages per rollout, specification of minimum ratios of super users per unit, and post implementation reinforcement rounds to ensure that training has been converted to regular use.

### Limitations and directions for future research

Several limitations should be noted. The cross-sectional design does not exclude causal inference and cannot exclude reverse or reciprocal relationships, despite the hypothesised directions theory consistent. Self-reported Likert scales were used to measure all constructs, which are prone to common method bias, but neither the single factor test by Harman nor the complete VIF of collinearity supported the notion of severe bias [25, 33]. Another weakness is representativeness: equal distribution across strata without post stratification weighting implies that estimates indicate the sample composition but not the entire EHC workforce and can be vicarious in the event that leadership or digital roles are over represented. Subsequent research ought to be longitudinal or multi-level, include objective performance measures, and integrate surveys with qualitative investigation to describe the mechanisms of leadership and knowledge processes during the process of digital transformation.

## Conclusion

This research explored the mediating role of knowledge transfer in the influence of transformational leadership on digital healthcare performance within the Eastern Health Cluster in Saudi Arabia with change management taken into consideration. The analysis conducted on PLS-SEM with 402 surveys of the staff indicated that transformational leadership had a strong positive impact on knowledge transfer and digital performance, that knowledge transfer is a robust direct predictor of digital performance, and that knowledge transfer mediated the leadership-performance relationship partially.

The results suggest a moderate explanation: transformational leadership seems to be able to direct digital healthcare performance by the direct organisational action and an indirect path that functions via an enhanced knowledge exchange regarding digital tools. The results add to the body of leadership and digital health research because they help to reduce uncertainties on the fact that the main mechanism of translating transformational leadership into digital healthcare performance in a large integrated health system is knowledge transfer. They also educate the implementation of Vision 2030 by implying that investment in technologies should be accompanied by leadership practices and structured knowledge sharing routines.

By the cross-sectional design, self-report measurement and the disproportionate stratified, non-weighted sampling, the conclusions should be viewed as associative and situation-specific, as opposed to population representative, cause and effect assertions. Nevertheless, as it is suggested to managers and policymakers in the Saudi Arabian setting and beyond, long-term digital performance enhancement is more probable when leadership development and knowledge transfer infrastructure is balanced to facilitate learning, coordination and workflow redesign across facilities.

## Supplementary Information

Below is the link to the electronic supplementary material.

Supplementary Material 1

Supplementary Material 2

## Data Availability

The datasets generated and analysed during the current study contain organisational and potentially identifiable information and are therefore not publicly available. De identified aggregated data may be made available from the corresponding author on reasonable request, subject to Eastern Health Cluster and institutional ethical approvals.

## Abbreviations

TL: Transformational leadership
CM: Change management
KT: Knowledge transfer
DHP: Digital healthcare performance
EHC: Eastern health cluster
PLS SEM: Partial least squares structural equation modeling
AVE: Average variance extracted
CR: Composite reliability
