# Supplementary material for: The mediating role of knowledge transfer in the relationship between transformational leadership and digital healthcare performance in the Eastern Health Cluster, Saudi Arabia
Source: BMC Health Serv Res. 2026 Apr 7;26:702. doi: 10.1186/s12913-026-14425-1 (PMC13181989; doi:10.1186/s12913-026-14425-1)
Supplement: Supplementary file 1 — Supplementary Material 1 [file 12913_2026_14425_MOESM1_ESM.docx]

# Supplementary File – English Version of the Questionnaire

## Transformational Leadership (TL)

TL1. My leader articulates a clear vision for digital transformation.

TL2. My leader motivates staff to achieve digital health goals.

TL3. My leader encourages new ideas to improve health services.

TL4. My leader promotes innovation in healthcare processes.

TL5. My leader considers individual staff needs in digital change.

TL6. My leader communicates expectations clearly.

TL7. My leader inspires a shared sense of purpose.

TL8. My leader fosters collaboration within the team.

TL9. My leader supports continuous learning.

TL10. My leader demonstrates confidence during change initiatives.

## Knowledge Transfer (KT)

KT1. Knowledge is shared openly among staff.

KT2. Staff collaborate to solve work-related problems.

KT3. Employees receive the information necessary to perform tasks.

KT4. There is clear documentation of digital health processes.

KT5. Staff share lessons learned from digital transformation.

KT6. Knowledge is transferred effectively between departments.

KT7. Training materials are accessible to all employees.

KT8. Mentoring or peer support helps in knowledge transfer.

KT9. Knowledge gained is applied to improve performance.

KT10. Management encourages knowledge sharing.

## Digital Healthcare Performance (DHP)

DHP1. Digital systems improve patient care quality.

DHP2. Digital health tools enhance service efficiency.

DHP3. Staff productivity has improved due to digital systems.

DHP4. Digital applications reduce administrative workload.

DHP5. Patient experience improved with digital services.

DHP6. Digital documentation is accurate and reliable.

DHP7. Digital systems support clinical decision-making.

DHP8. Digital tools facilitate timely access to patient data.

DHP9. Digital systems reduce medical errors.

DHP10. Digital performance supports organizational goals.

## Change Management (CM)

CM1. Employees are informed about changes in a timely manner.

CM2. Management provides reasons for digital transformation.

CM3. Staff are involved in planning digital initiatives.

CM4. Training is provided to adapt to new digital systems.

CM5. Employees receive adequate support during change.

CM6. Resistance to change is addressed effectively.

CM7. Communication about change is clear and consistent.

CM8. Leadership supports successful change implementation.

CM9. There are systems to monitor progress during change.

CM10. The organization adapts quickly to changes.
