# Supplementary material for: The mediating role of knowledge transfer in the relationship between transformational leadership and digital healthcare performance in the Eastern Health Cluster, Saudi Arabia
Source: BMC Health Serv Res. 2026 Apr 7;26:702. doi: 10.1186/s12913-026-14425-1 (PMC13181989; doi:10.1186/s12913-026-14425-1)
Supplement: Supplementary file 2 — Supplementary Material 2 [file 12913_2026_14425_MOESM2_ESM.pdf]

Appendices

Appendix A: Ethical Approval Letter

CLU0030_25_03_09_NEW APR_EXP.doc.pdf

Appendix B: Supplementary Table S1. Measurement Items, Provenance, and Adaptation Status

Construct	Item code	Source	Adopted or adapted	Adaptation notes (contextualisation)
Transformational leadership	TL1	batista foguett et al. (2021)	Adapted	Reworded to reflect leadership behaviours during digital health transformation within EHC
	TL2	batista foguett et al. (2021)	Adapted	Contextualised to digital vision, digital tools, and service redesign expectations
	TL3	batista foguett et al. (2021)	Adapted	Contextualised to ethical role modelling and support during digital implementation
	TL4	batista foguett et al. (2021)	Adapted	Contextualised to motivating staff adoption of new digital workflows
	TL5	batista foguett et al. (2021)	Adapted	Contextualised to intellectual stimulation around digital improvement and innovation
	TL6	batista foguett et al. (2021)	Adapted	Contextualised to individualised consideration during digitally enabled change
	TL7	batista foguett et al. (2021)	Adapted	Contextualised to communication clarity around digital health priorities
	TL8	batista foguett et al. (2021)	Adapted	Contextualised to encouraging reflection on how digital tools improve care quality
	TL9	batista foguett et al. (2021)	Adapted	Contextualised to coaching and staff support for digital capability building

	TL10	batista foguett et al. (2021)	Adapted	Contextualised to inspiring collective commitment to digital transformation goals
Knowledge transfer	KT1	Birasnav et al. (2013)	Adapted	Reworded to cover knowledge exchange about digital systems and routines
	KT2	Birasnav et al. (2013)	Adapted	Contextualised to sharing lessons learned from digital tools implementation
	KT3	Birasnav et al. (2013)	Adapted	Contextualised to use of guidelines and codified digital knowledge
	KT4	Liu and Li (2018)	Adapted	Contextualised to team based exchange and shared digital workflow learning
	KT5	Liu and Li (2018)	Adapted	Contextualised to peer support and problem solving for digital use cases
	KT6	Tuyen (2025)	Adapted	Contextualised to autonomy, engagement, and routine knowledge sharing about digital work
	KT7	Tuyen (2025)	Adapted	Contextualised to participation in training and applying learned digital practices
	KT8	Birasnav et al. (2013)	Adapted	Contextualised to cross unit learning and dissemination within a cluster model
	KT9	Liu and Li (2018)	Adapted	Contextualised to unit level exchange that improves shared understanding of digital tasks
	KT10	Tuyen (2025)	Adapted	Contextualised to applying transferred knowledge to improve practice outcomes

Digital healthcare performance (DHP)	DHP1	Al Kuwaiti et al. (2018)	Adapted	Reworded to reflect staff perceived impact of digital tools on care quality and coordination
	DHP2	Al Kuwaiti et al. (2018)	Adapted	Contextualised to timeliness of information and point of care decision support
	DHP3	Al Kuwaiti et al. (2018)	Adapted	Contextualised to patient centred service improvements enabled by digital tools
	DHP4	Kludacz Alessandri et al. (2025)	Adapted	Contextualised to integration of digital tools into clinical and managerial routines
	DHP5	Kludacz Alessandri et al. (2025)	Adapted	Contextualised to efficiency and workflow improvement through digital intensity
	DHP6	Kludacz Alessandri et al. (2025)	Adapted	Contextualised to interoperability and coordination across settings in a cluster model
	DHP7	Al Kuwaiti et al. (2018)	Adapted	Contextualised to data availability and documentation quality enabled by digital systems
	DHP8	Kludacz Alessandri et al. (2025)	Adapted	Contextualised to analytics and data use supporting performance improvement
	DHP9	Al Kuwaiti et al. (2018)	Adapted	Contextualised to communication and care continuity enabled by digital platforms
	DHP10	Kludacz Alessandri et al. (2025)	Adapted	Contextualised to overall staff perceived digital performance capability across routines
Change management	CM1	Choi and Ruona (2011)	Adapted	Reworded to reflect communication and support for digital change initiatives

	CM2	Choi and Ruona (2011)	Adapted	Contextualised to clarity of rationale for digital implementation
	CM3	Self et al. (2007)	Adapted	Contextualised to stakeholder involvement in digital projects and workflow redesign
	CM4	Self et al. (2007)	Adapted	Contextualised to training adequacy and implementation support for digital tools
	CM5	Choi and Ruona (2011)	Adapted	Contextualised to follow through and reinforcement during digital change
	CM6	Choi and Ruona (2011)	Adapted	Contextualised to leadership communication consistency during digital rollout
	CM7	Self et al. (2007)	Adapted	Contextualised to managing resistance and concerns related to digital transformation
	CM8	Self et al. (2007)	Adapted	Contextualised to resources and time provided to adapt to digital work processes
	CM9	Choi and Ruona (2011)	Adapted	Contextualised to evaluation and feedback loops during digital change
	CM10	Self et al. (2007)	Adapted	Contextualised to sustained support and alignment of digital change actions

Appendix C: Demographic characteristics of respondents (N = 402)

Attributes	Categories	Frequency	Percentage
Gender	Male	209	52
	Female	193	48
Age Groups	18-24 years	3	1
	25-34 years	84	21
	35-44 years	197	49
	45-54 years	101	25
	55-64 years	17	4
Level of Education	High School diploma or equivalent	28	7
	Bachelor's Degree	195	48
	Master' degree	123	31
	PhD	56	14
Job Position	Clinicians and Health informatics professionals	60	15
	Department heads	24	6
	EHC leader	23	5.5
	Finance and budget managers	2	0.5
	Hospital administrators	10	2.5
	Information technology staff e.g. (HIM, IT)	27	7
	IT or information systems managers	8	2
	Managers e.g. (EHC team, Hospital administrators)	153	38
	Medical directors	8	2
	Nursing directors	8	2
	Quality improvement and patient safety leaders	8	2
	Support Staff e.g. (Medical technicians, assistants and technologists)	55	13
Experience	0-5 Years	49	12.3
	6-10 Years	62	15.5
	11-15 Years	96	23.9
	16-20 Years	121	29.9
	More than 20 years	74	18.3

Appendix D: Descriptive Statistics For Key Study Constructs

Descriptive Statistics	Descriptive Statistics	Descriptive Statistics	Descriptive Statistics	Descriptive Statistics	Descriptive Statistics
	N	Minimum	Maximum	Mean	Std. Deviation
Average Score of Transformational Leadership	402	1.40	4.70	3.7134	.87159
Average Score of Change Management	402	1.20	5.00	3.3823	.80181
Average Score of Knowledge Transfer	402	1.10	5.00	3.6525	.97439
Average Score of Digital Healthcare Performance	402	1.60	4.70	3.6657	.79648

Appendix E: Internal Consistency Reliability of Study Constructs

Constructs	Cronbach's Alpha	No. of Items
Transformational Leadership	0.941	10
Change Management	0.960	10
Knowledge Transfer	0.950	10
Digital Healthcare Performance	0.920	10
Overall tool	0.965	40
